# Synthesis of Reduced Graphene Oxide-Modified LiMn_0.75_Fe_0.25_PO_4_ Microspheres by Salt-Assisted Spray Drying for High-Performance Lithium-Ion Batteries

**DOI:** 10.1038/srep26686

**Published:** 2016-05-25

**Authors:** Myeong-Seong Kim, Hyun-Kyung Kim, Suk-Woo Lee, Dong-Hyun Kim, Dianbo Ruan, Kyung Yoon Chung, Sang Hyun Lee, Kwang Chul Roh, Kwang-Bum Kim

**Affiliations:** 1 Department of Material Science and Engineering, Yonsei University, 134 Shinchon-dong, Seodaemoon-gu, Seoul 120-749, Republic of Korea; 2 Department of Materials Science and Metallurgy, University of Cambridge, 27 Charles Babbage Road, Cambridge CB3 0FS, UK; 3 Center for Energy Convergence Research, Korea Institute of Science and Technology, Hwarangno 14-gil 5, Seongbuk-gu, Seoul 136-791, Republic of Korea; 4 SkyChem, A-304 Keumkang IT Tower, 215, Galmachiro, Jungwon-gu, Seongnam, 462-901, Republic of Korea; 5 Energy Efficient Materials Team, Energy & Environmental Division, Korea Institute of Ceramic Engineering & Technology, 101 Soho-ro, Jinju-si, Gyeongsangnam-do, 660-031, Republic of Korea

## Abstract

Microsized, spherical, three-dimensional (3D) graphene-based composites as electrode materials exhibit improved tap density and electrochemical properties. In this study, we report 3D LiMn_0.75_Fe_0.25_PO_4_/reduced graphene oxide microspheres synthesized by one-step salt-assisted spray drying using a mixed solution containing a precursor salt and graphene oxide and a subsequent heat treatment. During this process, it was found that the type of metal salt used has significant effects on the morphology, phase purity, and electrochemical properties of the synthesized samples. Furthermore, the amount of the chelating agent used also affects the phase purity and electrochemical properties of the samples. The composite exhibited a high tap density (1.1 g cm^−3^) as well as a gravimetric capacity of 161 mA h g^−1^ and volumetric capacity of 281 mA h cm^−3^ at 0.05 C-rate. It also exhibited excellent rate capability, delivering a discharge capacity of 90 mA h g^−1^ at 60 C-rate. Furthermore, the microspheres exhibited high energy efficiency and good cyclability, showing a capacity retention rate of 93% after 1000 cycles at 10 C-rate.

As calls to replace fossil fuels in automobiles increase, lithium-ion batteries (LIBs) have come to be regarded as the most effective and practical devices for doing so^1^,^2^. In order to meet the demand for LIBs with high energy and power densities, the development of suitable electrode material designs is critical. To achieve this goal, nanostructured designs, including zero-dimensional (0D; nanoparticles), one-dimensional (1D; nanowires and nanotubes), two-dimensional (2D; nanosheets), and three-dimensional (3D; hollow spheres and core-shell structures) structures, have been suggested. Among these nanostructured designs, composites of nanosized active materials and conductive carbon materials have been attracting great attention because most electrode materials have very low electrical conductivity^3^,^4^,^5^,^6^.

Among the various conductive carbon materials available, graphene sheets or reduced graphene oxide (rGO) have attracted considerable attention owing to their high electronic/thermal conductivity, and large surface area^7^,^8^,^9^,^10^. Recently, graphene- or rGO-based composite materials have been studied extensively, as these materials exhibit significantly improved electrochemical properties^3^,^11^,^12^,^13^. However, most reported studies on these composite materials for LIBs have only focused on the gravimetric electrochemical properties. Because graphene sheets or rGO have a large surface area, 2D graphene- or rGO-based composites have low tap densities (<0.8 g cm^−3^). Because of this problem, their volumetric energy densities are limited^14^,^15^,^16^. Further, because the volumetric properties are also important with respect to LIBs, improvements in the tap density of 2D graphene- or rGO-based composites are highly desirable.

In order to overcome this limitation of 2D graphene-based composite materials, micrometer-scale 3D graphene-based composite materials composed of nanosized active materials captured within microsized, spherical 3D graphene networks have been suggested^17^,^18^. These microspherical 3D graphene-based composite materials have several advantages: First, the tap density of 3D graphene-based composites is higher than that of nanoparticles or 2D graphene-based composites^19^,^20^. Second, a continuous electron path exists within the 3D graphene-based microspheres; this is effective in decreasing the charge-transfer resistance^3^. Third, the numerous pores present between the nanosized active material and the graphene sheets can allow the deep penetration of the electrolyte ions, thereby improving the Li-ion accessibility^14^,^19^,^21^. Although a few studies have focused on the synthesis of microspherical 3D graphene-based composites, the methods used in these studies have been time-consuming multistep ones involving the spray drying of pre-synthesized nanoparticles on graphene oxide sheets. In addition, the reported salt-assisted spray-drying method has mostly been used to synthesize simple binary transition metal oxides (M_x_O_y_)^22^,^23^,^24^,^25^. Therefore, a simple method for synthesizing microspherical 3D graphene-based composites of multiple transition metal oxides is highly desirable.

In this study, we report the one-step fabrication of a microsized, spherical 3D graphene-based composite consisting of carbon-coated LiMn_0.75_Fe_0.25_PO_4_ nanoparticles captured within a 3D graphene microspherical structure using a salt-assisted spray drying method. Although LiMn_0.75_Fe_0.25_PO_4_ has come to be regarded as a promising cathode material for LIBs, the use of LiMn_0.75_Fe_0.25_PO_4_ has been impeded by its extremely poor electrochemical properties, which are attributable to its intrinsically low electronic conductivity and Li^+^ diffusion rate and by the fact that it undergoes Mn dissolution during cycling^4^,^19^,^21^,^26^,^27^. Therefore, the microspherical 3D graphene-based composite structure should be ideal for improving the electrochemical properties of the LiMn_0.75_Fe_0.25_PO_4_ electrode material. The composite was synthesized by a simple one-pot salt-assisted spray-drying process using a mixture of a solution of chelated metal salts (citric acid-Fe^+2^ and citric acid-Mn^+2^), a LiH_2_PO_4_ salt solution, and an aqueous dispersion of GO and a subsequent heat treatment. The 3D LiMn_0.75_Fe_0.25_PO_4_/rGO microspheres synthesized in this manner exhibited a high tap density, high specific capacity, excellent rate capability, and superior cycling stability as a cathode material for LIBs. Further, this method can potentially be used to synthesize other graphene-based composite materials simply by changing the composition of the precursor solution used.

## Results

**Figure 1:**
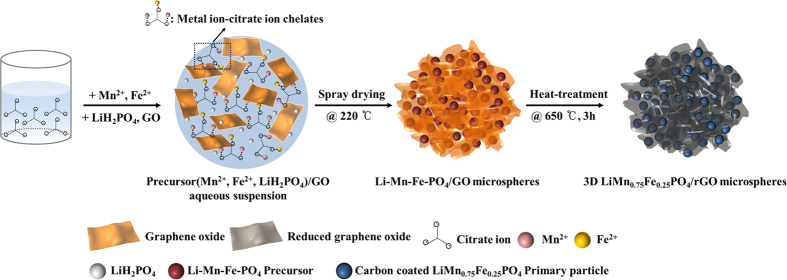
Schematic illustration showing the formation of the 3D LiMn_0.75_Fe_0.25_PO_4_/rGO microspheres.

The formation of the 3D LiMn_0.75_Fe_0.25_PO_4_/rGO microspheres is schematically illustrated in Fig. 1. First, citric acid is dissolved in deionized (DI) water. In the aqueous solution, the citric acid ionizes into citrate ions (C_6_H_5_O_7_^3−^)^28^. When the manganese and iron salts are dissolved in the aqueous citric acid solution, the metal ions present (Mn^2+^ and Fe^x+^ (x = 2 or 3)) bond with the citrate ions (C_6_H_5_O_7_^3−^) through electrostatic interactions. After the GO dispersion has been mixed with the above-mentioned solution, metal ion-citrate ion chelates are dispersed uniformly within the GO sheets. Next, an aqueous LiH_2_PO_4_ solution is added to the mixed solution and the solution is spray dried. During this process, it is nebulized, forming fine droplets as it leaves the nozzle. When these droplets are subsequently dried in pre-heated air, the GO sheets spontaneously assemble on the surfaces of the droplets, owing to their amphiphilicity, and shrink to form microspheres^29^,^30^,^31^. Thus, microspherical Li-Mn-Fe-PO_4_/GO composite precursors are obtained by the spray-drying process. After a subsequent heat treatment, phase-pure 3D LiMn_0.75_Fe_0.25_PO_4_/rGO microspheres are formed, while a uniform carbon coating is formed on the surfaces of the LiMn_0.75_Fe_0.25_PO_4_ primary particles by the carbonization of the citrate ions attached to the metal ions^32^,^33^. Thus, 3D LiMn_0.75_Fe_0.25_PO_4_/rGO microspheres composed of carbon-coated primary nanoparticles and rGO sheets could be successfully synthesized by using a one-pot salt-assisted spray-drying method and a subsequent heat treatment.

**Figure 2:**
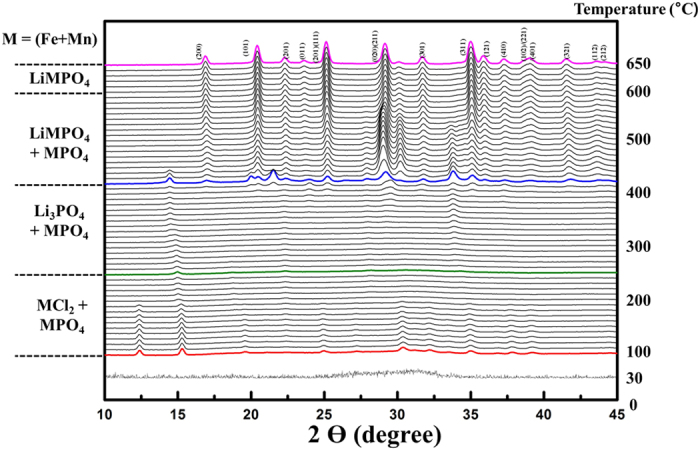
*In-situ* time-resolved X-ray diffraction (TR-XRD) pattern of the Li-Mn-Fe-PO_4_/GO precursor prepared using the metal chlorides when heated to 650 °C in an inert atmosphere.

The results of TGA and a DSC analysis of the Li-Mn-Fe-PO_4_/GO microspheres prepared using the metal chlorides are shown in Fig. S1. These results confirm that the heat treatment at 650 °C in Ar was sufficient for synthesizing phase-pure LiMn_0.75_Fe_0.25_PO_4_^34^,^35^. To further study the phase evolution of the 3D LiMn_0.75_Fe_0.25_PO_4_/rGO microspheres during the heat treatment, *in-situ* TR-XRD analysis was employed. Most previous analyses of the formation of materials with an olivine structure have been performed using *ex-situ* XRD^35^. However, *ex-situ* XRD analyses cannot characterize the intermediates formed by the reactions that occur during the heat-treatment process. For this reason, we employed *in-situ* TR-XRD analysis to characterize in real time the reactions occurring during the heat treatment in an inert atmosphere. The results of the *in-situ* TR-XRD analysis are shown in Fig. 2. The temperature during the *in-situ* TR-XRD analysis was increased from 100 to 650 °C while using the dried Li-Mn-Fe-PO_4_/GO microspheres synthesized using the metal chlorides. After the spray-drying process, the precursor did not exhibit any diffraction peaks, indicating that it was amorphous. After the precursor had been dried at 100 °C in an oven, in order to evaporate the remaining water, its XRD pattern indicated that it had become crystalline, with the pattern corresponding to that of a mixed phase consisting of MCl_2_ (M = Fe and Mn) and LiH_2_PO_4_. As the temperature was increased to 280 °C, the peaks observed at approximately 12, 30, and 35° disappeared. This indicated the formation of MPO_4_ and Li_3_PO_4_ phases. When the temperature reached 450 °C, an olivine LiMPO_4_ phase was formed, with a MPO_4_ phase also being present. This result was in keeping with that of the TGA, shown in Fig. S1. Upon heating, the diffraction peaks corresponding to the MPO_4_ phase decreased gradually in intensity. Finally, phase-pure LiMn_0.75_Fe_0.25_PO_4_ was formed at 650 °C in the inert atmosphere. This confirmed that a heat treatment at 650 °C in an inert atmosphere is suitable for obtaining phase-pure 3D LiMn_0.75_Fe_0.25_PO_4_/rGO microspheres^35^.

**Figure 3:**
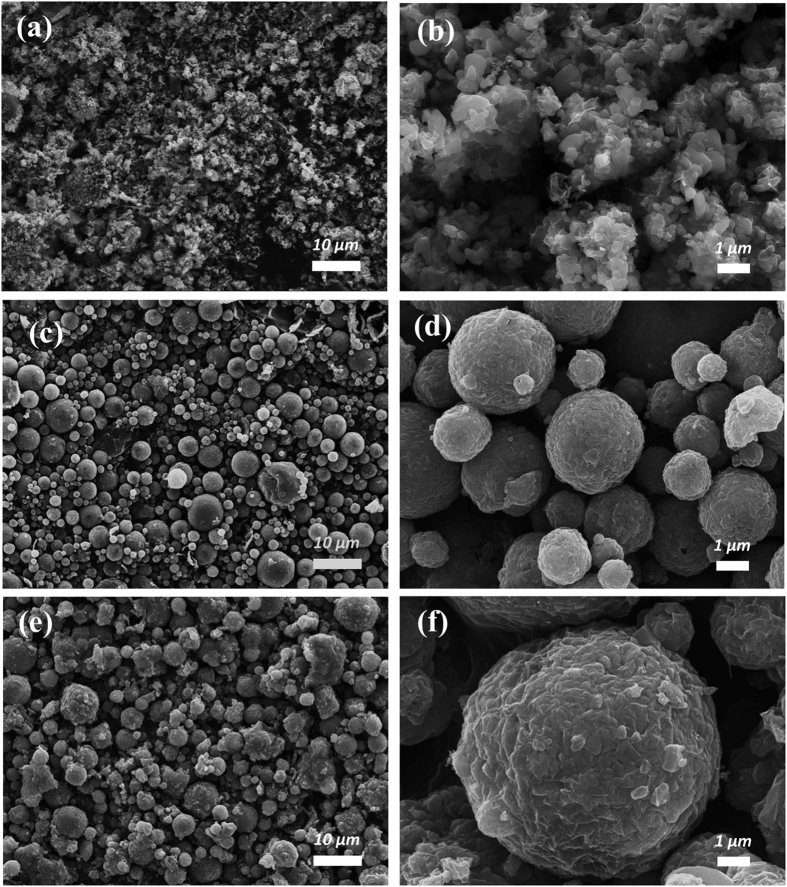
FE-SEM images of 3D LiMn_0.75_Fe_0.25_PO_4_/rGO microspheres prepared using different metal salts: (**a,b**) SO_4__LMFP, (**c,d**) NO_3__LMFP, and (**e,f**) Cl_2__LMFP.

The morphologies of the samples prepared using different metal salts, as determined by FE-SEM, are shown in Fig. 3. As can be seen from Fig. 3(a,b), SO_4__LMFP consists of a mixture of LiMn_0.75_Fe_0.25_PO_4_ nanoparticles and rGO sheets. The size of the LiMn_0.75_Fe_0.25_PO_4_ nanoparticles is approximately 200–500 nm and they are uniformly mixed with the rGO sheets. When the metal sulfates are used in the precursor, various gases such as H_2_O, O_2_, and SO_2_ evolve during the spray-drying process. Because the vapor pressures of O_2_ and SO_2_ are high, these gases can cause a high pressure within the droplet system. The spherical shape of the droplets is thus distorted by the high pressure induced by the evolved gases^36^. Therefore, the metal sulfates were not suitable for forming the microspherical composite.

Interestingly, when the metal nitrates and the metal chlorides were used, the resulting samples, namely, NO_3__LMFP and Cl_2__LMFP, exhibited spherical morphologies, as shown in Fig. 3(c–f). The size of the spherical secondary particles is approximately 2–7 μm. A high-magnification SEM image (Fig. 3(d–f)) revealed that the LiMn_0.75_Fe_0.25_PO_4_ nanoparticles were wrapped in rGO sheets. Thus, we concluded that the choice of the metal salt used is a key factor for obtaining composites with a microspherical morphology. In addition, it is known that rGO sheets retard particle growth during heat treatments^37^. If the spheres were to be synthesized without rGO (as was the case for rGO-free LiMn_0.75_Fe_0.25_PO_4_/C), the size of the primary particles would not be on the nanoscale (Fig. S3).

**Figure 4:**
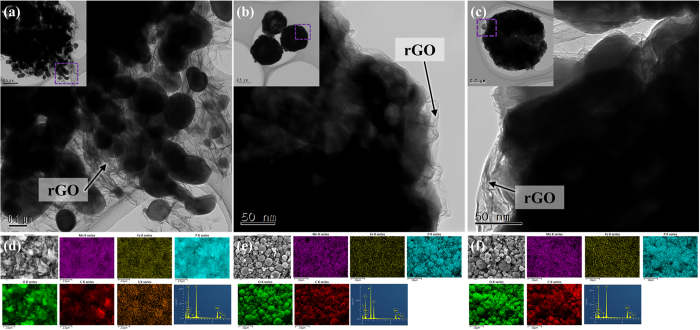
TEM images of 3D LiMn_0.75_Fe_0.25_PO_4_/rGO microspheres synthesized using different metal salts: (**a**) SO_4__LMFP, (**b**) NO_3__LMFP, and (**c**) Cl_2__LMFP. Energy-dispersive X-ray spectroscopy (EDS) elemental maps of the 3D LiMn_0.75_Fe_0.25_PO_4_/rGO microspheres: (**d**) SO_4__LMFP, (**e**) NO_3__LMFP, and (**f**) Cl_2__LMFP.

Figure 4(a–c) show TEM images of the samples synthesized using the different metal salts. As shown in Fig. 4(a), in the case of SO_4__LMFP, LiMn_0.75_Fe_0.25_PO_4_ nanoparticles with a size of 100–200 nm are uniformly dispersed on the surfaces of the rGO nanosheets. However, as shown in Fig. 4(b,c), NO_3__LMFP and Cl_2__LMFP have a microspherical morphology, with the surfaces of the microspheres covered with the rGO sheets. The results of EDS elemental mapping of the samples are shown in Fig. 4(d–f). In the case of SO_4__LMFP (see Fig. 4(d)), the elements Fe, Mn, P, O, and C were distributed over the composite particles. Unfortunately, the element S was also detected as an impurity all over SO_4__LMFP. Because S has very low electrical conductivity (~10^−30^ S cm^−1^), the electrochemical properties of the SO_4__LMFP may be inferior^38^. In the cases of NO_3__LMFP and Cl_2__LMFP (see Fig. 4(e,f)), the elements present Fe, Mn, P, O, and C were homogeneously distributed all over the 3D LiMn_0.75_Fe_0.25_PO_4_/rGO microspheres, and no impurity was observed.

**Figure 5:**
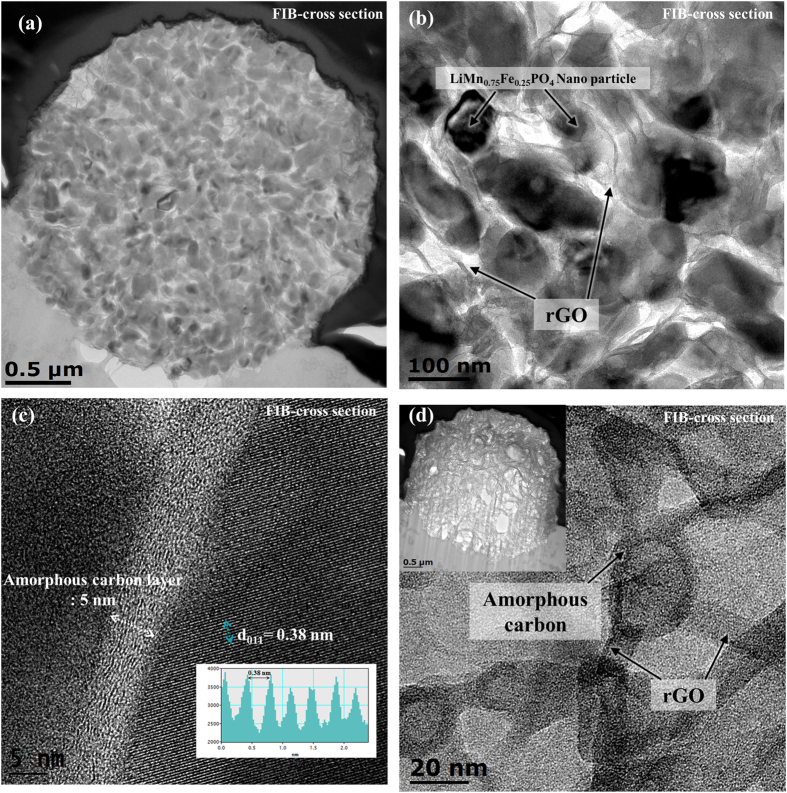
(**a,b**) Cross-sectional TEM images, (**c**) Cross-sectional HR-TEM image of Cl_2__LMFP. (**d**) Cross-sectional TEM images of the carbon network in Cl_2__LMFP.

Figure 5(a,b) show cross-sectional TEM images of FIB-etched Cl_2__LMFP. It can be seen that the LiMn_0.75_Fe_0.25_PO_4_ primary nanoparticles and the rGO nanosheets were evenly dispersed not only near the surfaces of the microspheres but also within the microspheres themselves. This structure can provide an effective electron pathway at the surfaces as well as within the interiors of the microspheres. Moreover, this 3D structure was porous and contained interconnected nanopores with diameters smaller than 50 nm. The nanopores present in the 3D LiMn_0.75_Fe_0.25_PO_4_/rGO microspheres allowed the electrolyte ions to penetrate deep, resulting in improved Li-ion accessibility.

Figure 5(c) shows a cross-sectional HR-TEM image of Cl_2__LMFP. It can be seen that the LiMn_0.75_Fe_0.25_PO_4_ primary nanoparticles were well crystallized and had a d-spacing of 0.37 nm, which corresponded to the (011) plane^4^,^39^. The amorphous carbon from the citric acid was uniformly coated on the surface of each primary nanoparticle in a thickness of 4–5 nm. This layer of amorphous carbon effectively reduced the charge-transfer resistance, resulting in improvements in the electrochemical performance of the secondary particles. In addition, the carbon coating prevented direct contact between the nanoparticles and the electrolyte, further improving the electrochemical and cycling stabilities^40^.

In order to investigate the carbon network composed of rGO and amorphous carbon in the 3D LiMn_0.75_Fe_0.25_PO_4_/rGO microspheres, the LiMn_0.75_Fe_0.25_PO_4_ primary nanoparticles were selectively removed by immersing the microspheres in an HCl solution. A cross-sectional TEM image of the 3D carbon network is shown in Fig. 5(d). Even after the LiMn_0.75_Fe_0.25_PO_4_ primary nanoparticles had been removed, the secondary particles maintained their spherical morphology. This suggested that the rGO sheets were distributed homogeneously all over the microspheres^29^. In addition, the amorphous carbon formed by the carbonization of the citric acid could be seen clearly. This unique 3D carbon structure composed of rGO sheets and amorphous carbon could effectively decrease the charge-transfer resistance.

**Figure 6:**
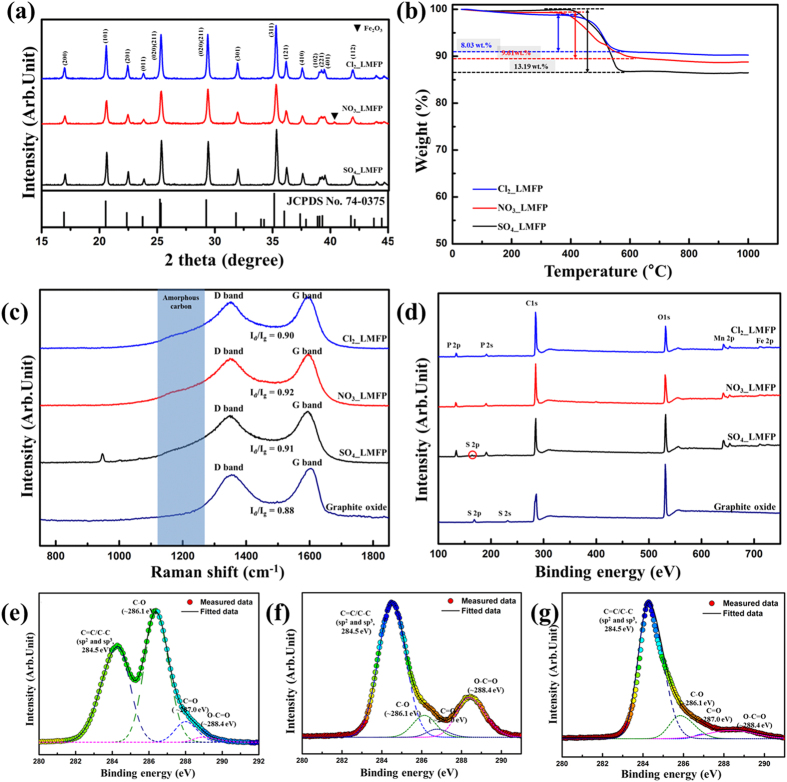
(**a**) XRD patterns, (**b**) TGA curves in ambient air, (**c**) Raman spectra, and (**d**) full-scale XPS of the 3D LiMn_0.75_Fe_0.25_PO_4_/rGO microspheres synthesized using the different metal salts. XPS C1s spectra of (**e**) GO, (**f**) the Li-Mn-Fe-PO_4_/GO composite precursors, and (**g**) Cl_2__LMFP.

Figure 6(a) shows the XRD patterns of the 3D LiMn_0.75_Fe_0.25_PO_4_/rGO microspheres synthesized using the different metal salts. The XRD peaks of SO_4__LMFP and Cl_2__LMFP corresponded to those of impurity-free olivine-structured materials (JCPDS Card No. 74-0375). However, the XRD peaks of NO_3__LMFP suggested that it had an impurity phase such as Fe_2_O_3_. Owing to Fe^3+^ salts such as Fe(NO_3_)_3_ being used in the synthesis, it seems that NO_3__LMFP contained a small amount of an impurity such as Fe_2_O_3_. Because it is well known that the impurities present in olivine-structured materials significantly degrade their electrochemical performances, the electrochemical performance of NO_3__LMFP was probably inferior to those of SO_4__LMFP and Cl_2__LMFP. Furthermore, to investigate the effects of the amount of chelating agent, different amounts of citric acid were used in the precursors in which metal chlorides were used as the salts. Figure S2 shows that although the amount of citric acid did not affect the formation of spherical morphologies of the products, it had a significant effect on the phase purity of the products. When the concentration of citric acid used was lower than 1 M, impurities such as Fe_2_O_3_ were formed in the samples. These impurities degraded the electrochemical performances of the samples.

To investigate the amount of Fe_2_O_3_ impurity in the composites, we examined the valence states of Fe ion in the 3D LiMn_0.75_Fe_0.25_PO_4_/rGO microspheres, based on the XPS Fe 2p_3/2_ spectra (Fig. S5). Because the valence states of Fe ions in the composite and impurity were different, i.e., +2 and +3, respectively, it was possible to infer the amounts of Fe_2_O_3_ impurity in the composites using the XPS Fe 2p_3/2_ spectra. The Fe 2p_3/2_ spectra of the composites showed peaks typical of Fe^+2^ ions (at ~709.5 and ~714.2 eV) and Fe^+3^ ions (at ~712.2 eV)^41^. The percentages of Fe^+3^ ions in the total amounts of Fe ions in SO_4__LMFP, NO_3__LMFP, and Cl_2__LMFP determined from the XPS Fe 2p_3/2_ spectra were 3.62%, 12.39%, and 2.33%, respectively. These results indicate that the amount of Fe_2_O_3_ impurity in NO_3__LMFP was ~3.10%. (The amount of Fe ion in the composite * the amount of Fe^+3^ ion = 0.25 * 12.39% = 3.10%).

The LiMn_0.75_Fe_0.25_PO_4_ nanoparticle content in the 3D LiMn_0.75_Fe_0.25_PO_4_/rGO microspheres was determined using TGA performed in air; the results are shown in Fig. 6(b). The weights of the samples decreased as the temperature was increased from 400 to 600 °C, indicating that the carbon or the sulfur in the samples underwent oxidation. The weight losses of SO_4__LMFP, NO_3__LMFP, and Cl_2__LMFP were 13.19, 9.61, and 8.03 wt%, respectively. Because SO_4__LMFP contained carbon in the form of rGO, in addition to amorphous carbon and sulfur, the weight loss of SO_4__LMFP was larger than those of NO_3__LMFP and Cl_2__LMFP. The carbon and sulfur contents of the composite were accurately determined using elemental analysis (EA); the results are listed in Table S3. EA showed that the carbon contents of SO_4__LMFP, NO_3__LMFP, and Cl_2__LMFP were 8.51, 8.13, and 7.88 wt%, respectively; the carbon contents of the samples were similar to each other. The only difference among the composites was the sulfur contents. Among the composites, the sulfur was only detected in SO_4__LMFP. The sulfur content of SO_4__LMFP was about 2.76 wt%. Furthermore, referring to the TGA result for rGO-free LiMn_0.75_Fe_0.25_PO_4_/C, as shown Fig. S4, the rGO and amorphous carbon contents in Cl_2__LMFP were approximately 3 and 5 wt%, respectively.

Figure 6(c) shows the Raman spectra used to analysis the structure of the carbon in the samples. The Raman spectra of all the samples exhibited two prominent peaks related carbon; these corresponded to the D (arising from structural imperfections in the A_1g_ mode) and G bands (arising from the first-order scattering of the E_2g_ mode). Interestingly, the Raman spectra of the composites contained features observed in the spectra of both the rGO-free LiMn_0.75_Fe_0.25_PO_4_/C and graphite oxide (Fig. S4). This result suggested that heterostructured carbons such as rGO and amorphous carbon were present in the 3D LiMn_0.75_Fe_0.25_PO_4_/rGO microspheres^42^,^43^. SO_4__LMFP (I_d_/I_g_ = 0.91), NO_3__LMFP (I_d_/I_g_ = 0.92), and Cl_2__LMFP (I_d_/I_g_ = 0.90) clearly show higher D-to-G band intensity ratios than does graphite oxide (I_d_/I_g_ = 0.88), indicating that the reduction of the GO nanosheets in the composites decreased the average size of the sp^2^ domains^44^. In addition, the band at approximately 1000 cm^−1^, which is attributable to the A_g_ mode of ν_1_ and the antisymmetric stretching modes of the PO^4−^ anions, was seen only in the Raman spectrum of SO_4__LMFP, because the penetration depth of the light during Raman scattering is very small^45^,^46^.

Figure 6(d) shows the full-scale XPS spectra for graphite oxide and the synthesized composites. The constituent elements of graphite oxide as detected by XPS were C, O, and S. The amount of sulfur in the graphite oxide sample was approximately 6.15 wt% and was an impurity from sulfuric acid. NO_3__LMFP and Cl_2__LMFP contained the elements Fe, Mn, P, O, and C and no impurities. However, SO_4__LMFP clearly showed peaks related to S as an impurity element. This result was in keeping with the EDS elemental maps shown in Fig. 4. In addition, the intensity of the O1s peak in the case of the composites was lower than that of graphite oxide, indicating that the graphite oxide in the composites was successfully reduced to rGO^47^,^48^.

In order to investigate the degree of reduction of GO in the samples, XPS C1s analyses were performed, as shown in Fig. 6(e–g). The spectrum of GO (Fig. 6(e)) contained typical components related to the C=C/C-C (sp^2^ and sp^3^, ~284.5 eV), C-O (hydroxyl and epoxy, ~286.1 eV), C=O (carbonyl, ~287.0 eV), and O-C = O (carboxyl, ~288.4 eV) groups^48^,^49^. The relative atomic percentages of sp^2^/sp^3^ carbons and oxygen-containing functional groups in these materials are listed in Table S2. It can be seen that GO contained 42.4% sp^2^/sp^3^ carbon components and 57.1% oxygen-containing functional groups such as C-O, C = O, and O-C = O. In comparison, before the heat treatment, the Li-Mn-Fe-PO_4_/GO composite precursors (Fig. 6(f)) contained 66.9% sp^2^/sp^3^ carbon components and 33.1% oxygen-containing functional groups. This confirmed that GO was partially reduced during the spray-drying process owing to drying by the heated air. In addition, after the heat treatment, Cl_2__LMFP (Fig. 6(g)) contained 77.4% sp^2^/sp^3^ carbon components and 22.6% oxygen-containing functional groups. In particular, the O-C = O carboxyl functional group was almost completely removed during the heat treatment. Consequently, these results confirmed that GO was reduced to rGO through spray drying and the subsequent heat treatment.

**Figure 7:**
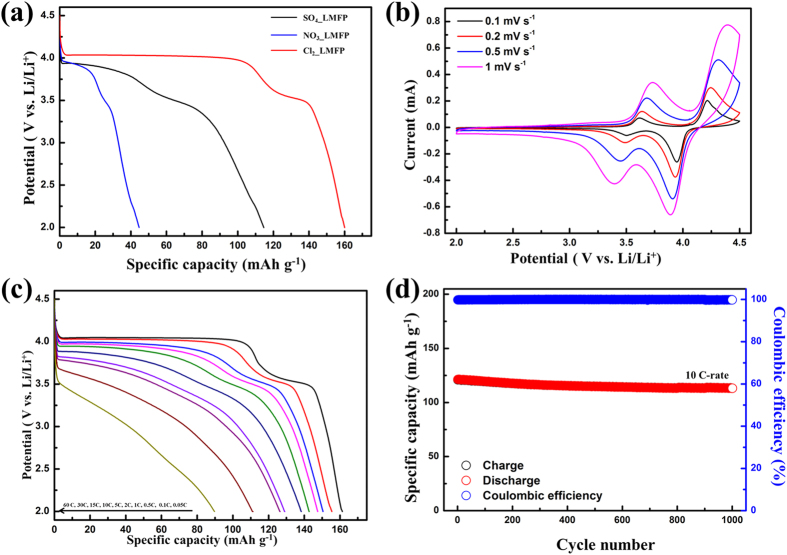
(**a**) Galvanostatic discharge curves of the LiMn_0.75_Fe_0.25_PO_4_/rGO microspheres synthesized using the different metal salts at 0.05 C. (**b**) Cyclic voltammograms of Cl_2__LMFP obtained at scan rates of 0.1, 0.2, 0.5, and 1.0 mV s^−1^. (**c**) Galvanostatic discharge curves of Cl_2__LMFP obtained at current rates of 0.05–60 C-rate. (**d**) Long-term cyclability of Cl_2__LMFP at 10 C-rate.

Figure 7(a) shows the galvanostatic discharge curves of the samples prepared using the different metal salts at 0.05 C-rate. The discharge capacities of SD_NO_3_ and SD_SO_4_ were 45 mA h g^−1^ and 114 mA h g^−1^, respectively. Because SD_NO_3_ contained impurities such as Fe_2_O_3_, its discharge capacity was very low. It has been reported that impurities severely degrade the electrochemical performance of such materials^26^. In addition, the discharge capacity of SO_4__LMFP at a low rate was also very low, owing to the presence of elemental of S in the sample, which resulted in extremely low electrical conductivity. SD_Cl_2_ showed the highest discharge capacity, which was 161 mAh g^−1^ and corresponded to 94% of the theoretical capacity. The charge/discharge profile of SD_Cl_2_ exhibiting its gravimetric and volumetric characteristics is shown in Fig. S6. The volumetric discharge capacity of SD_Cl_2_ at 0.05 C-rate was 281 mA h cm^−3^. This is similar to the previously reported value for LiMn_0.75_Fe_0.25_PO_4_ without rGO^26^,^33^,^50^. This result is interesting because it is well known that graphene-based composite materials exhibit very low volumetric energy densities. Further, this result is attributable to the high tap density (1.1 g cm^−3^) of SD_Cl_2_. To investigate the effect of rGO on the electrochemical properties of the samples, the electrochemical performances of SD_Cl_2_ were compared with those of the rGO-free LiMn_0.75_Fe_0.25_PO_4_/C, as shown Fig. S7. The electrochemical performances of SD_Cl_2_ were better than those of the rGO-free LiMn_0.75_Fe_0.25_PO_4_/C. These results mean that the rGO was effective in improving the electrochemical performances, owing to its high electronic conductivity. Furthermore, when 1 M citric acid was used, SD_Cl_2_ exhibited the best electrochemical performance (Fig. S8).

Figure 7(b) shows the cyclic voltammograms for SD_Cl_2_, obtained at scan rates of 0.1, 0.2, 0.5, and 1.0 mV s^−1^. The CV at 0.1 mVs^−1^ shows clearly two sets of current peaks, at approximately 3.5 V and 4.0 V; these corresponded to the redox reactions of Fe^2+^/Fe^3+^ and Mn^2+^/Mn^3+^, respectively^51^. Interestingly, the shape of the voltammogram remained unchanged with an increase in the potential scan rates to 1 mV s^−1^. Because the shape of the peak in a CV curve reflects the kinetics of Li^+^ insertion/deinsertion, it can be deduced that the prepared SD_Cl_2_ sample showed improved electrochemical reaction kinetics of Li^+^ insertion/deinsertion^52^,^53^.

Figure 7(c) shows the discharge curves of SD_Cl_2_ at 0.05–60 C-rates. The discharge capacities of the microspheres were 161, 155, 150, 147, 142, 138, and 129 mA h g^−1^ at C-rates of 0.05, 0.1, 0.5, 1, 2, 5, and 10, respectively. In addition, the discharge capacity measured at an extremely high rate of 60 C was approximately 90 mA h g^−1^, which is 56% of the specific capacity at 0.05 C-rate. The excellent rate capabilities of the microspheres can be attributed to the unique carbon structure of the hybrid material, which consisted of highly conductive rGO sheets and a surface coating of amorphous carbon; these served as electrically conductive channels for the LiMn_0.75_Fe_0.25_PO_4_ nanoparticles. In addition, the porous structure of SD_Cl_2_ allowed the Li^+^ ions to penetrate deep into the secondary particles, resulting in the full electrochemical utilization of the microspheres. The electrochemical properties of SD_Cl_2_ were compared to those published in the literature (see Fig. S9). The rate capabilities of SD_Cl_2_ prepared in this study were better than or similar to those reported previously, indicating that the microspheres exhibited excellent high-rate capabilities.

Figure 7(d) shows the long-term cycling stability after 1000 cycles at 10 C-rate of SD_Cl_2_. After 1000 cycles, the capacity retention rate was 93%. In addition, the composite electrode also exhibited a stable coulombic efficiency of up to 99% after 1000 cycles. This result indicated that SD_Cl_2_ exhibited excellent structural integrity during repeated cycling. This could be attributed to their 3D microspherical structure as well as the surface carbon coating, which prevented direct contact with the electrolyte.

**Figure 8:**
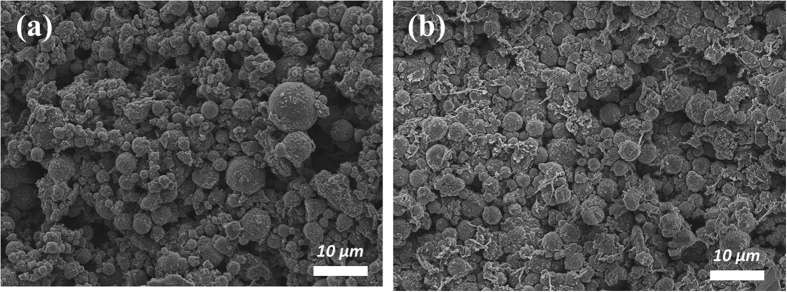
SEM images of (**a**) an as-prepared electrode based on SD_Cl_2_ and (**b**) the electrode after 1000 cycles at a charge/discharge rate of 10 C-rate.

In order to investigate the structural stability of SD_Cl_2_ after long-term cycling tests, SEM analyses were performed on an electrode of the microspheres before and after cycling tests (Fig. 8(a,b), respectively). Even after 1000 cycles, the morphology of SD_Cl_2_ was remained. These results suggested that SD_Cl_2_ exhibited good structural stability during electrochemical reactions.

To further understand the electrochemical behaviors of the samples, EIS analyses were performed at 4.04 V after 5 charge/discharge cycles at 0.1 C-rate. The results of the EIS analyses of the electrodes of the rGO-free LiMn_0.75_Fe_0.25_PO_4_/C and SD_Cl_2_ can be seen in Fig. S10. The Nyquist plots for the electrodes of both materials consisted of a compressed semicircle in the high/medium-frequency region and an inclined line in the low-frequency region; these were assignable to the charge-transfer resistance and the semi-diffusion of the Li ions into LiMn_0.75_Fe_0.25_PO_4_, respectively^49^. The electrode based on SD_Cl_2_ displayed a significantly lower charge-transfer resistance than that of the rGO-free LiMn_0.75_Fe_0.25_PO_4_/C electrode. This was owing to the unique 3D carbon-containing heterostructure of the microspheres, which improved electronic conductivity by allowing direct contact between the carbon-coated LiMn_0.75_Fe_0.25_PO_4_ nanoparticles and the rGO sheets.

## Discussion

In this study, 3D LiMn_0.75_Fe_0.25_PO_4_/rGO microspheres were successfully synthesized using a simple one-step salt-assisted spray-drying method and a subsequent heat treatment while employing a complexing agent. The complexing agent acted as both a reducing agent and the carbon source for the carbon coating. The 3D microspherical graphene-based composite consisted of 100 nm carbon-coated LiMn_0.75_Fe_0.25_PO_4_ nanoparticles entrapped within a 3D graphene structure. During this process, it was found that the choice of the metal salt used had a significant effect on the morphology, phase purity, and electrochemical properties of the obtained samples. Furthermore, the amount of the chelating agent used also affected the phase purity and electrochemical properties of the samples. The composite exhibited a high tap density and excellent electrochemical properties in terms of their gravimetric and volumetric specific capacity, rate capability, and cycling stability; this could be attributed to the surface carbon coating, the 3D structure consisting of nanoparticles integrated with graphene nanosheets, and the porous nature of the material. Furthermore, the proposed synthesis method should also be suitable for synthesizing other graphene-based electrode materials for various electrochemical energy storage and conversion devices.

## Methods

### Synthesis of 3D LiMn_0.75_Fe_0.25_PO/rGO microspheres using metal salts

As mentioned above, the 3D LiMn_0.75_Fe_0.25_PO_4_/rGO microspheres were prepared by a simple one-pot salt-assisted spray-drying process and a subsequent heat treatment. To determine the appropriate metal salt for this process, various salts, such as metal sulfates (MSO_4_), metal chlorides (MCl_2_), and metal nitrates (M(NO_3_)_x_) were tests (M = Fe, Mn). In a typical procedure, manganese salts (MnSO_4_ ∙ H_2_O(Aldrich), Mn(NO_3_)_2_ ∙ 4H_2_O(Aldrich), and MnCl_2_(Aldrich)) and iron salts (FeSO_4_ ∙ 7H_2_O(Aldrich), Fe(NO_3_)_3_ ∙ 9H_2_O(Aldrich), and FeCl_2_∙ 4H_2_O(Aldrich)) were dissolved in 10 ml of a 1 M aqueous solution of citric acid (Junsei Chemical). The citric acid acted as both the reducing agent as well as the carbon source for the carbon coatings. Next, 5 ml of a 1.2 M aqueous solution of LiH_2_PO_4_ (Aldrich) was added to the above-mentioned mixture. The Li:Mn:Fe:P molar ratio in the precursor was set to 1:0.75:0.25:1.

The GO used in this study was prepared from purified natural graphite powder (≤45 μm, Aldrich) using a modified Hummers’ method^49^. Once synthesized, the GO was exfoliated and dispersed in deionized water in a concentration of 1.0 mg ml^−1^ by ultrasonication to obtain a GO suspension. Next, 100 ml of this GO suspension was mixed homogeneously with the above-mentioned mixture under strong magnetic stirring. Finally, the mixed solution was spray dried. The temperature of the inlet air during the spray-drying process was maintained at 220 °C. The obtained product was then placed in a quartz tube in vacuum and heated to 100 °C for 6 h. The as-obtained composite precursors were then heat treated at 650 °C for 3 h in an Ar atmosphere; the heating rate was 5 °C min^−1^. The samples obtained using the metal sulfates, metal nitrates, and metal chlorides were denoted as SO_4__LMFP, NO_3__LMFP, and Cl_2__LMFP, respectively. In addition, for comparison, rGO-free LiMn_0.75_Fe_0.25_PO_4_/C was also prepared by the same procedure using only the metal chlorides; the only difference was that a GO dispersion was not added to the precursor solution.

### Synthesis of 3D LiMn_0.75_Fe_0.25_PO/rGO microspheres using different amounts of the chelating agent

In order to investigate the effects of the chelating agent, using only the metal chlorides, samples were prepared while employing citric acid in different amounts. To control the amount of citric acid used, the concentrations of citric acid used were 0.25, 0.5, 1, and 2 M. The 3D LiMn_0.75_Fe_0.25_PO_4_/rGO microspheres were synthesized using the above-described procedures. The samples obtained using 0.25, 0.5, 1, and 2 M citric acid were denoted as 0.25M_LMFP, 0.5M_LMFP, 1M_LMFP, and 2M_LMFP, respectively.

### Characterization

To investigate the phase evolution of the samples, *in-situ* time-resolved X-Ray diffraction (TR-XRD) analyses (R-AXIS IV++, Rigaku) were performed at the Korea Institute of Science and Technology (KIST). Approximately 3–4 mg of the sample being tested was loaded into a quartz capillary with an inner diameter of 0.7 mm. One end of this quartz capillary was open and the other was closed. After the sample had been loaded, Ar gas was injected into the capillary over a period of 1 h. Then, the open end of the capillary was closed completely. Next, the capillary was mounted on the thermal stage of the TR-XRD system. The TR-XRD patterns (~5 min for each XRD scan) of the sample were collected continuously as the sample was heated from room temperature to 650 °C at a rate of 2 °C min^−1^. Mo-Kα radiation with a wavelength of 0.7107 Å was used for the TR-XRD analyses. To allow for a ready comparison of the obtained results with those reported in the literature, the 2ϴ values were converted into values corresponding to Cu-Kα radiation (λ = 1.54 Å).

The morphologies of the samples were observed using field-emission scanning electron microscopy (FE-SEM) (JSM-7001F, JEOL Ltd.) and high-resolution transmission electron microscopy (HR-TEM) (JE-ARM 200F, JEOL Ltd.). The specimens for the cross-sectional TEM observations were prepared using a focused ion beam (FIB) (FEI Helios NanoLabTM 600). To measure the tap densities of the samples, the product being tested was placed in a small measuring cylinder and tapped repeatedly (1000 times) using a tap-density tester (TAP-2S, Logan Instruments). The crystalline phases of the samples were characterized through powder XRD analysis (40 kV, 20 mA; Rigaku) performed using Cu-Kα radiation (λ = 1.5406 Å). The thermal characteristics of the samples were investigated through thermogravimetric analysis (TGA) and differential scanning calorimetry (DSC) (STA 409 PC, Netzsch), which was performed at temperatures ranging from room temperature to 1000 °C; the heating and cooling rate was 10 °C min^–1^. Elemental analysis (EA, 2400 Series II, PerkinElmer) was performed to determine the amount of the carbon and the sulfur in the composites. In addition, the surfaces of the samples were analyzed using X-ray photoelectron spectroscopy (XPS) (15 kV, 150 W; ESCALAB 250, Thermo Electron Corporation). Raman spectroscopy (Jobin-Yvon LabRAM HR) was performed at room temperature using the conventional backscattering geometry and a liquid-N_2_-cooled charge-coupled device (CCD) multichannel detector. To investigate the compositions of the samples, inductively coupled plasma optical emission spectroscopy (ICP-OES) (OPTIMA 7300DV, Perkin Elmer) was used.

### Electrochemical measurements

The electrochemical properties of the samples were measured using CR2032-type coin cells, which used Li metal as the counter electrode, 1 M LiPF_6_ in a mixture of ethylene carbonate (EC)/ethyl methyl carbonate (EMC)/diethyl carbonate (DEC) in a volume ratio of 3:5:2 as the electrolyte, and polypropylene (Celgard 2400) as the separator. The working electrode was fabricated by mixing the samples, carbon black, and polyvinylidene fluoride (PVDF; Aldrich) in a weight ratio of 8:1:1. The resulting slurry was coated on an aluminum foil and dried in a vacuum oven at 90 °C for 24 h. Each working electrode had an area of 1.13 cm^2^, and the amount of the active material in each electrode was 3–4 mg cm^−2^. Cyclic voltammetry (CV) and galvanostatic charge/discharge tests were performed at voltages of 2.0–4.5 V (vs. Li/Li^+^) using a potentiostat/galvanostat (MPG2, Bio-Logic). Electrochemical impedance spectroscopy (EIS) was performed using an impedance analyzer (VMP2, Bio-Logic) at 4.04 V and an AC amplitude of 10 mV for frequencies ranging from 200 kHz to 10 mHz.

## Additional Information

**How to cite this article**: Kim, M.-S. *et al*. Synthesis of Reduced Graphene Oxide-Modified LiMn_0.75_Fe_0.25_PO_4_ Microspheres by Salt-Assisted Spray Drying for High-Performance Lithium-Ion Batteries. *Sci. Rep.* **6**, 26686; doi: 10.1038/srep26686 (2016).

## Supplementary Material

Supplementary Information
