# Corrigendum: Synthesis of Reduced Graphene Oxide-Modified LiMn_0.75_Fe_0.25_PO_4_ Microspheres by Salt-Assisted Spray Drying for High-Performance Lithium-Ion Batteries

**DOI:** 10.1038/srep29147

**Published:** 2016-07-11

**Authors:** Myeong-Seong Kim, Hyun-Kyung Kim, Suk-Woo Lee, Dong-Hyun Kim, Dianbo Ruan, Kyung Yoon Chung, Sang Hyun Lee, Kwang Chul Roh, Kwang-Bum Kim

Scientific Reports 6: Article number: 2668610.1038/srep26686; published online: 05252016; updated: 07112016

The Acknowledgements section in this Article is incomplete.

“This work was supported by the energy efficiency and resources grant (No: 20122010100140) of the Korea Institute of Energy Technology Evaluation and Planning (KETEP) funded by the Ministry of Knowledge Economy, Korean government. This work was also supported by POSCO and Business for Cooperative R&D between Industry, Academy, and Research Institute funded Korea Small and Medium Business Administration in 2015 (grant no. C0300716). This research was also supported by Basic Science Research Program through the National Research Foundation of Korea (NRF) funded by the Ministry of Science, ICT & Future Planning (2015R1A2A2A03006633).”

should read:

“This work was supported by the energy efficiency and resources grant (No: 20122010100140) of the Korea Institute of Energy Technology Evaluation and Planning (KETEP) funded by the Ministry of Knowledge Economy, Korean government. This work was also supported by POSCO and Business for Cooperative R&D between Industry, Academy, and Research Institute funded Korea Small and Medium Business Administration in 2015 (grant no. C0300716). This research was also supported by Basic Science Research Program through the National Research Foundation of Korea (NRF) funded by the Ministry of Science, ICT & Future Planning (2015R1A2A2A03006633). This work was also supported by the third Stage of Brain Korea 21 Plus Project in 2016.”

