# Supplementary material for: Synthesis of Reduced Graphene Oxide-Modified LiMn0.75Fe0.25PO4 Microspheres by Salt-Assisted Spray Drying for High-Performance Lithium-Ion Batteries
Source: Sci Rep. 2016 May 25;6:26686. doi: 10.1038/srep26686 (PMC4879626; doi:10.1038/srep26686)
Supplement: Supplementary Information [file srep26686-s1.doc]

Synthesis of Reduced Graphene Oxide-Modified LiMn_0.75_Fe_0.25_PO_4_ Microspheres by Salt-Assisted Spray Drying for High-Performance Lithium-Ion Batteries

Myeong-Seong Kim^a^, Hyun-Kyung Kim^a,b^, Suk-Woo Lee^a^, Dong-Hyun Kim^a,c^,Dianbo Ruan^a^, Kyung Yoon Chung^c^, Sang Hyun Lee^d^, Kwang Chul Roh^e*^ and Kwang-Bum Kim^a*^

^a^Department of Material Science and Engineering, Yonsei University, 134 Shinchon-dong, Seodaemoon-gu, Seoul 120-749, Republic of Korea

^b^Department of Materials Science and Metallurgy, University of Cambridge, 27 Charles Babbage Road, Cambridge CB3 0FS, UK

^c^Center for Energy Convergence Research, Korea Institute of Science and Technology, Hwarangno 14-gil 5, Seongbuk-gu, Seoul 136-791, Republic of Korea

^d^SkyChem, A-304 Keumkang IT Tower, 215, Galmachiro, Jungwon-gu, Seongnam, 462-901, Republic of Korea

^e^Energy Efficient Materials Team, Energy & Environmental Division, Korea Institute of Ceramic Engineering & Technology, 101 Soho-ro, Jinju-si, Gyeongsangnam-do, 660-031, Republic of Korea

**Supplementary Table**

**Table S1.** Atomic ratios of the samples, as determined by ICP-OES analysis.

| Sample | Li/P | Mn/P | Fe/P |
| --- | --- | --- | --- |
| SO4_LMFP | 0.97 | 0.74 | 0.26 |
| NO3_LMFP | 0.95 | 0.73 | 0.27 |
| Cl2_LMFP | 1.00 | 0.74 | 0.26 |

**Table S2 XPS C1s peak positions and relative atomic percentages of sp^2^, sp^3^, and oxygen-containing functional groups for graphite oxide and Cl2_LMFP before and after the heat treatment.**

| Sample | C=C/C-C | C-O | C=O | O-C=O |
| --- | --- | --- | --- | --- |
| Graphite oxide | 42.4 % | 48.4 % | 6.9 % | 1.8 % |
| Li-Mn-Fe-PO_4_/GO  (before heat treatment) | 66.9 % | 9.2 % | 3.2 % | 20.7 % |
| Cl2_LMFP  (after heat treatment) | 77.4 % | 9.6 % | 3.0 % | 8.5 % |

**Table S3.** Elemental Analysis Results for SO4_LMFP, NO3_LMFP, and Cl2_LMFP.

|  | Results (wt%) | | | |
| --- | --- | --- | --- | --- |
| Samples | Carbon | Hydrogen | Nitrogen | Sulfur |
| SO4_LMFP | 8.51 | 0.05 | 0.02 | 2.76 |
| NO3_LMFP | 8.13 | 0.10 | 0.01 | - |
| Cl2_LMFP | 7.88 | 0.05 | 0.03 | - |

**Supplementary Figures**

**
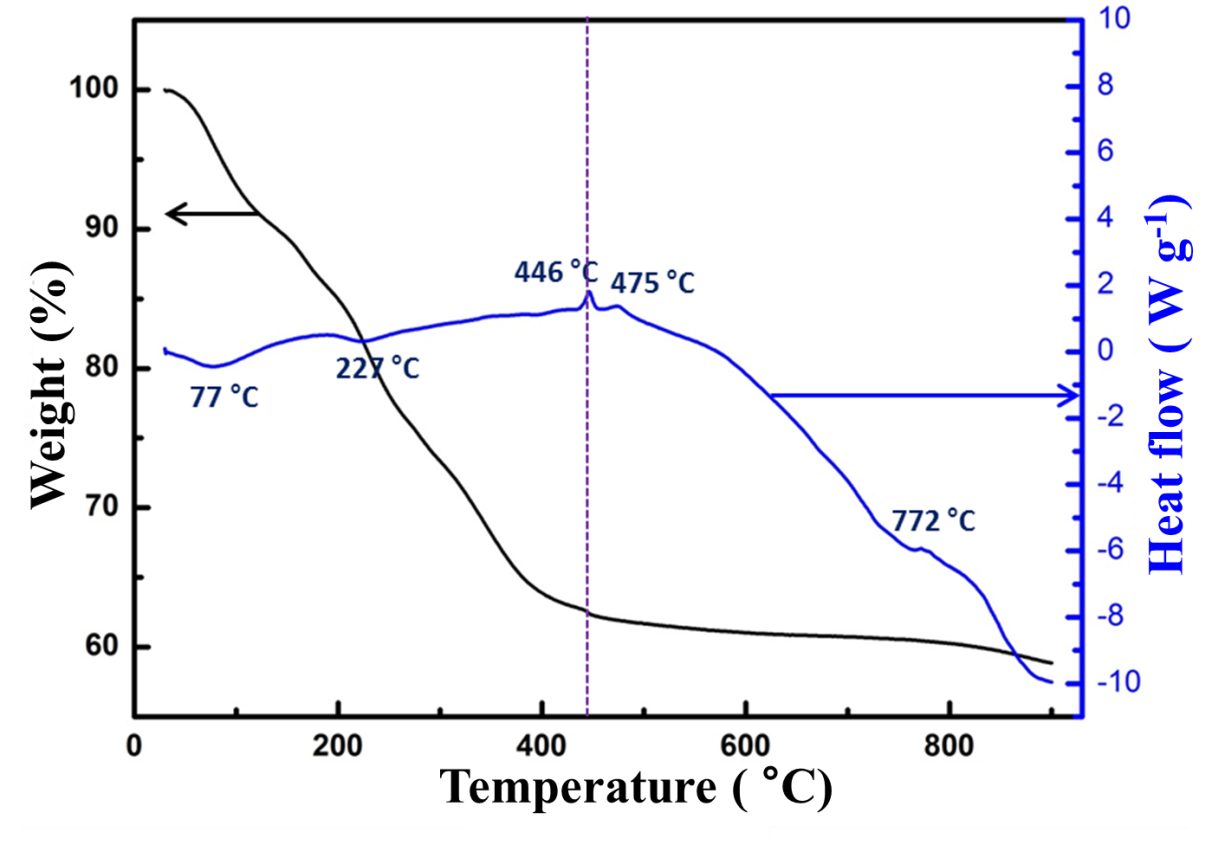
**

**Figure S1.** TG-DSC curves of the Li-Mn-Fe-PO_4_/GO microspheres prepared by using the metal chlorides.

The TGA and DSC analysis were performed at temperatures ranging from room temperature to 1000 °C in Ar; the heating rate was 10 °C min^–1^. Two endothermic peaks were observed in the DSC curve, at 77 and 227 °C; these corresponded to the decomposition of the oxygen-containing functional groups of GO, the decomposition of LiH_2_PO_4_, and the removal of the lattice water of FeCl_2_ ·4H_2_O. Although no other peaks were observed in the DSC curves for temperatures lower than 446 °C, there was a continual loss in weight, as determined from the TG curve. This loss in weight indicated that FeCl_2_ and MnCl_2_ decomposed and reacted with the decomposed LiH_2_PO_4_. The exothermic peak at 446 °C was indicative of the formation of olivine-phase LiMn_0.75_Fe_0.25_PO_4_. The weight decreased slightly as the temperature was increased from 446 to 772 °C; this was owing to the further nucleation and growth of the LiMn_0.75_Fe_0.25_PO_4_ grains. However, it has been reported that heat treatments at temperatures greater than 700 °C induce Fe-related impurities in olivine-structured materials. Thus, we performed the heat treatment at 650 °C, which was sufficient for the synthesis of phase-pure LiMn_0.75_Fe_0.25_PO_4_.

**
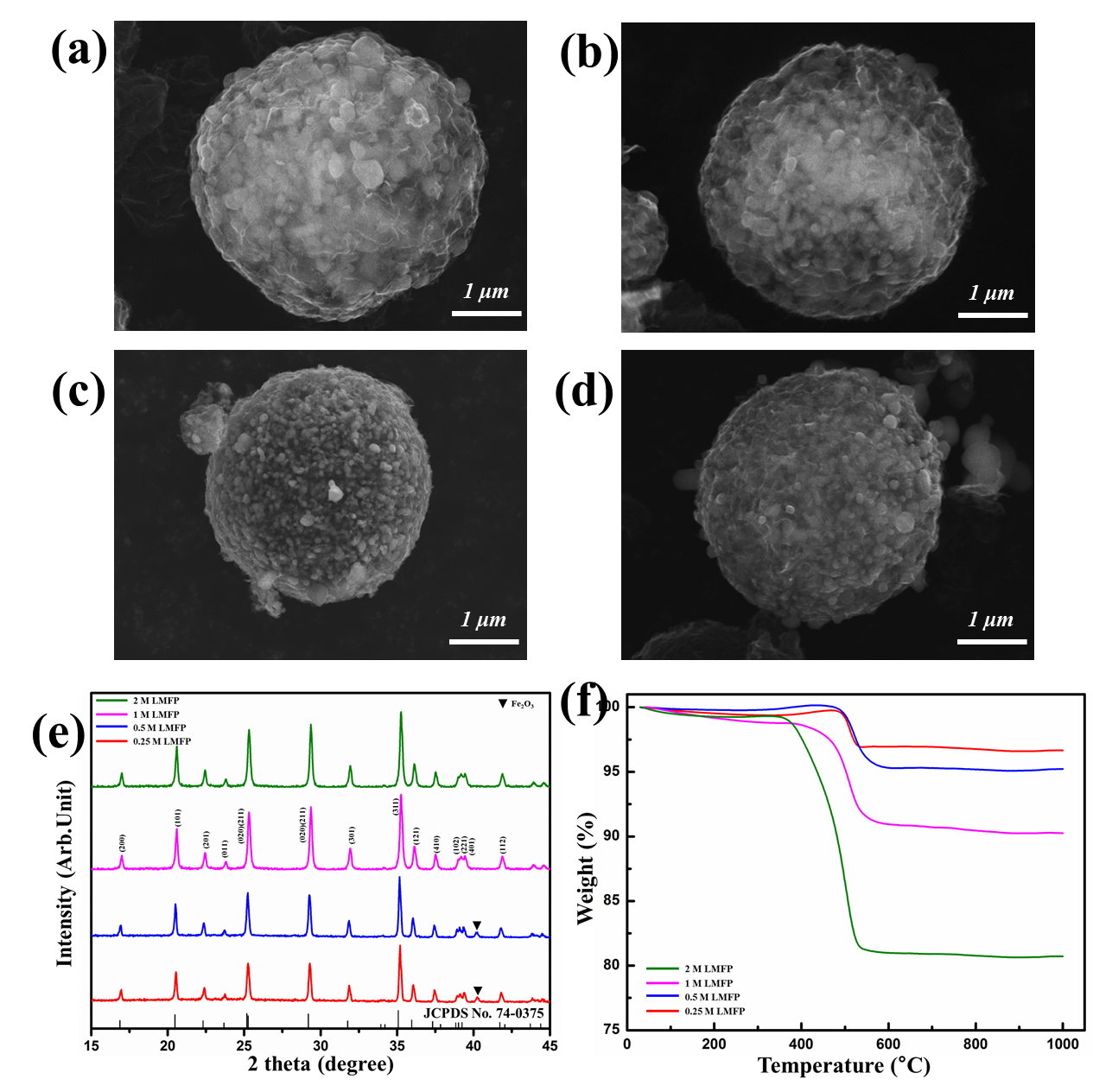
**

**Figure S2.** FE-SEM images of the 3D LiMn_0.75_Fe_0.25_PO_4_/rGO microspheres synthesized using different amounts of the chelating agent: (a) 0.25 M LMFP, (b) 0.5 M LMFP, (c) 1 M LMFP, and (d) 2 M LMFP. (e) XRD patterns and (f) TGA analysis results of the 3D LiMn_0.75_Fe_0.25_PO_4_/rGO microspheres synthesized using different amounts of the chelating agent.


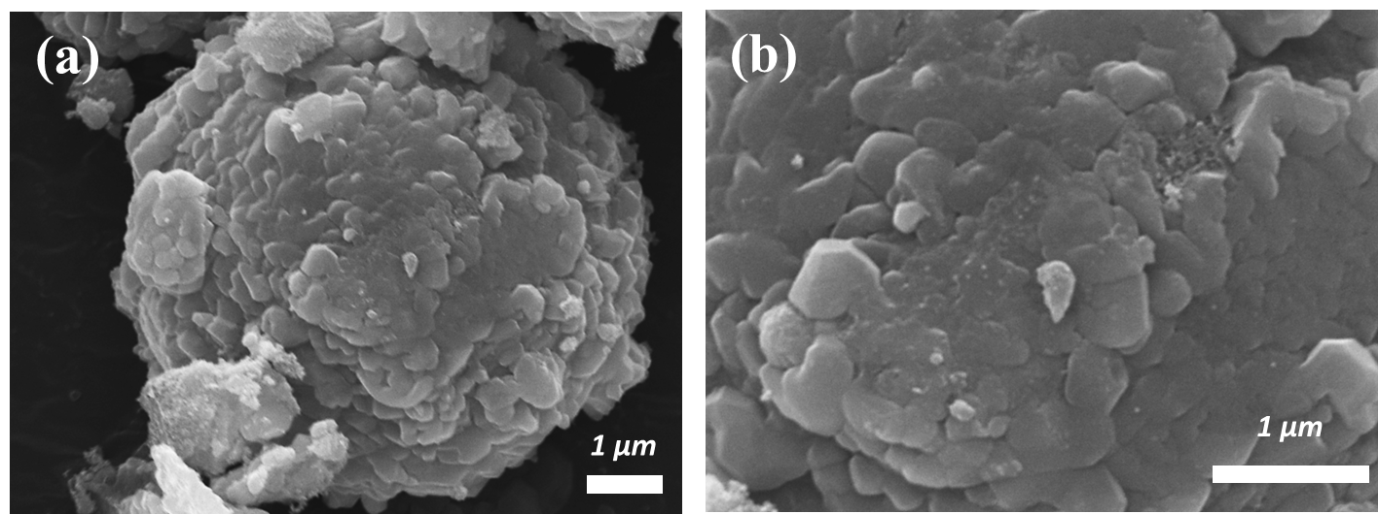


**Figure S3.** FE-SEM images of rGO-free LiMn_0.75_Fe_0.25_PO_4_/C prepared by using the metal chlorides: (a) low-magnification and (b) high-magnification images.


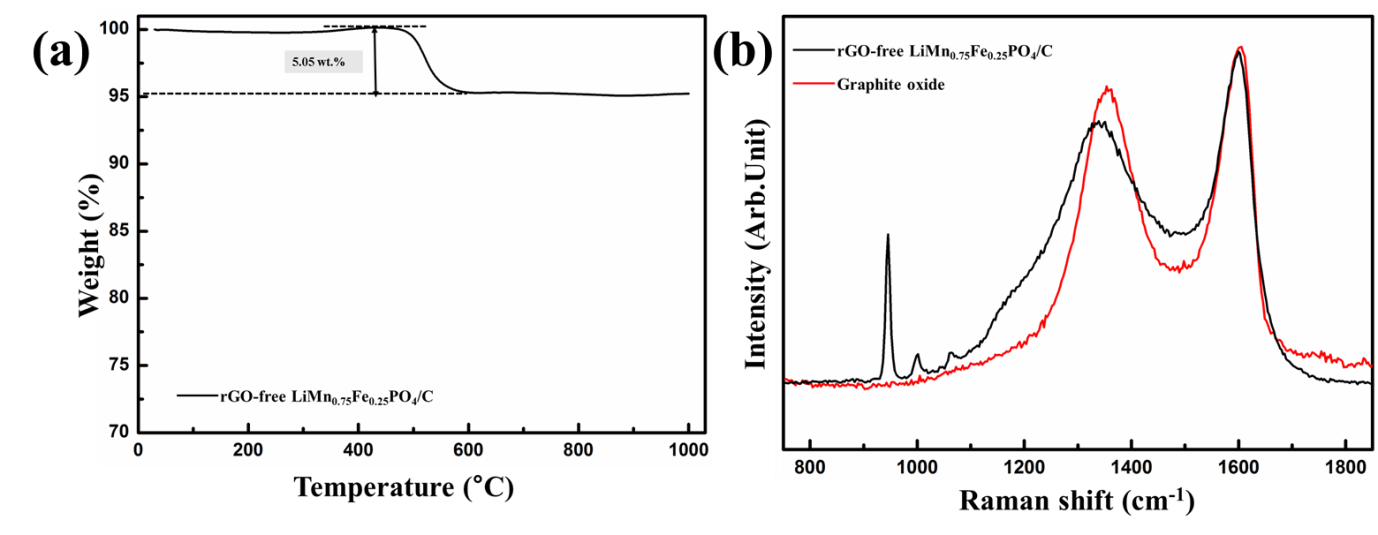


**Figure S4.** (a) TGA analysis of rGO-free LiMn_0.75_Fe_0.25_PO_4_/C prepared by using the metal chlorides. (b) Raman spectra of graphite oxide and rGO-free LiMn_0.75_Fe_0.25_PO_4_/C.

**
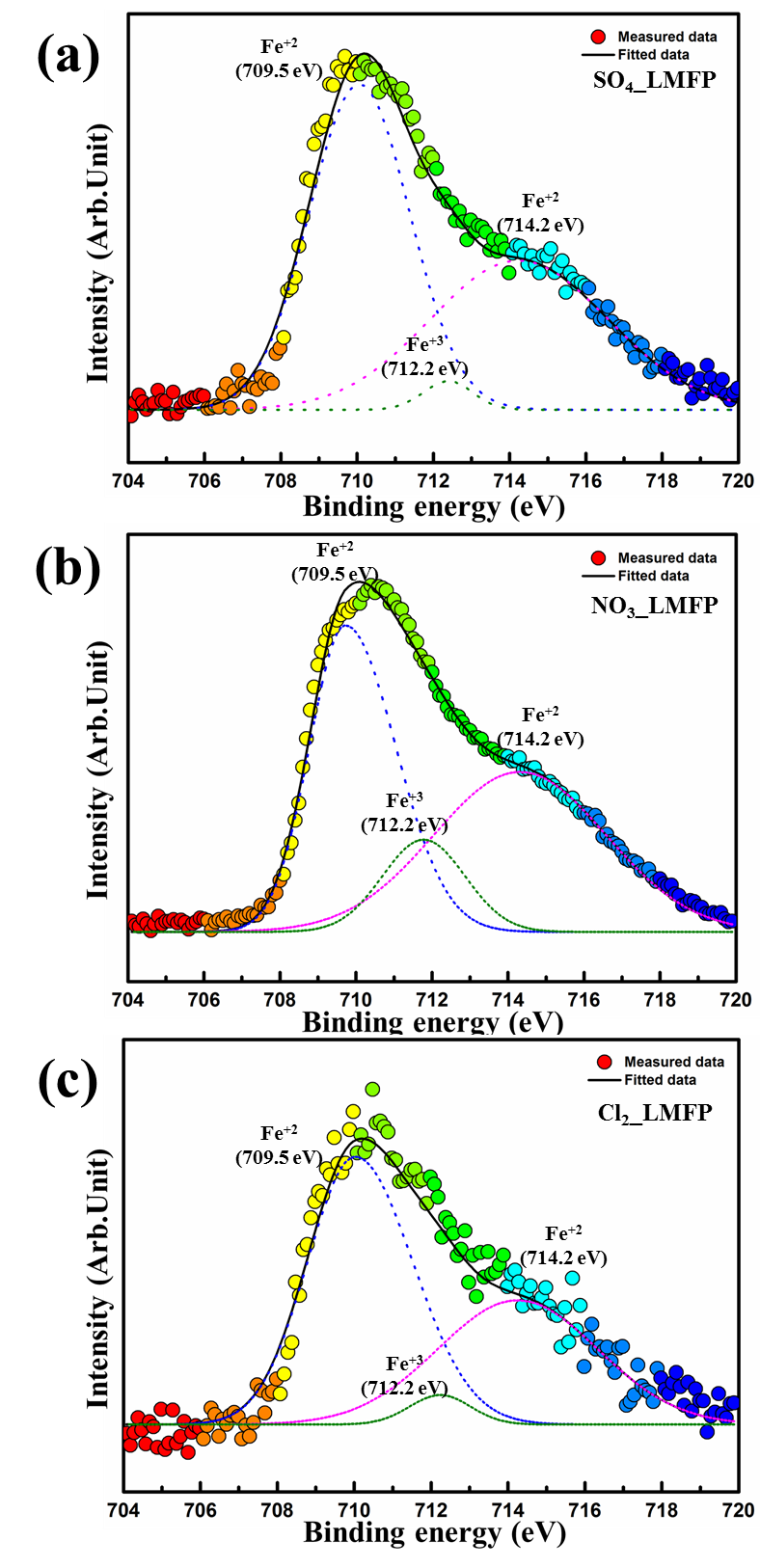
**

**Figure S5.** XPS Fe 2p_3/2_ spectra of (a) SO4_LMFP, (b) NO3_LMFP, and (c) Cl2_LMFP


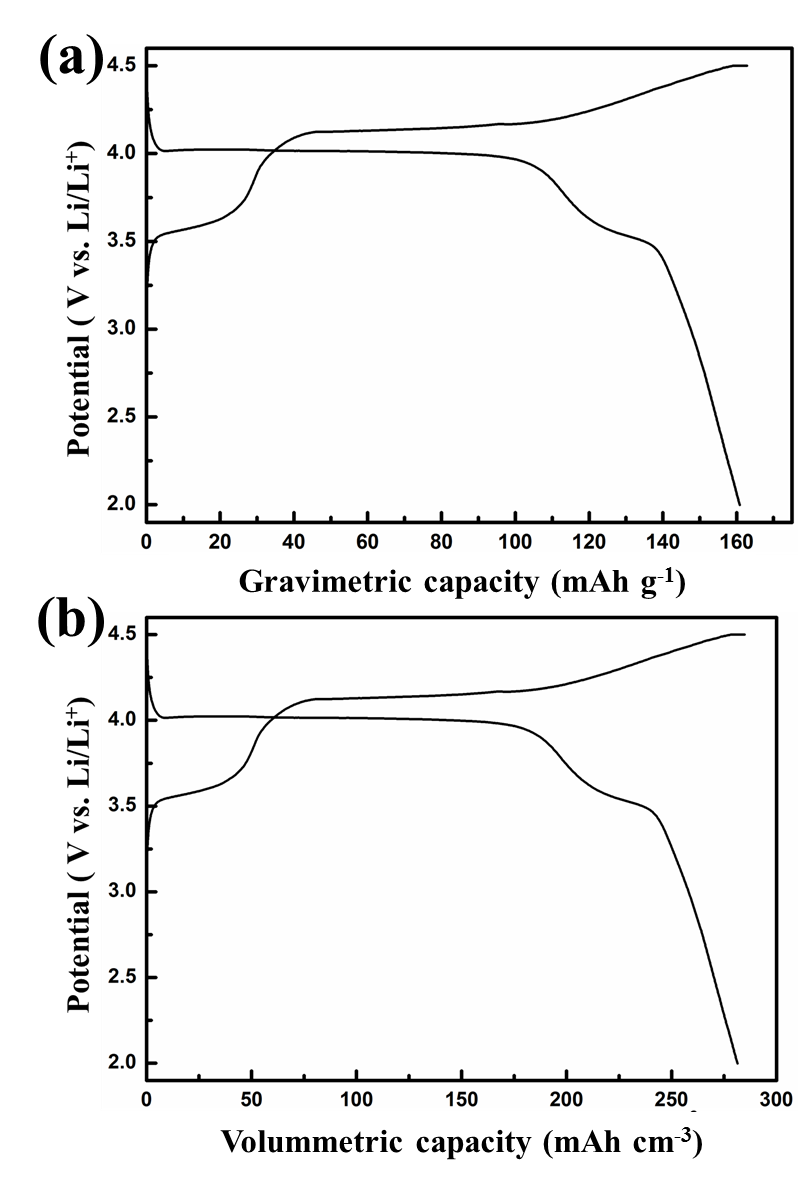


**Figure S6.** Charge-discharge curves of Cl2_LMFP at a C-rate of 0.05 in terms of the (a) gravimetric capacity and (b) volumetric capacity.


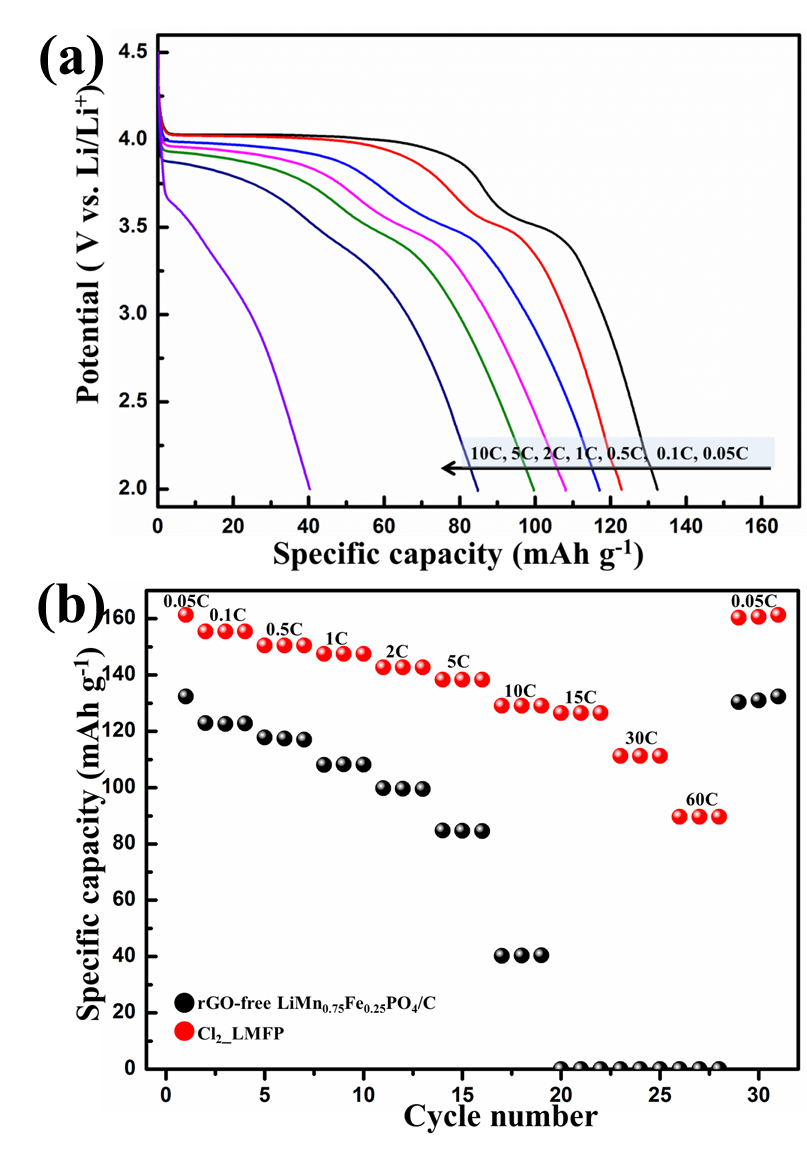


**Figure S7.** (a) Galvanostatic discharge curves at various C-rates of rGO-free LiMn_0.75_Fe_0.25_PO_4_/C and (b) gravimetric capacities of rGO-free LiMn_0.75_Fe_0.25_PO_4_/C and Cl2_LMFP for rates of 0.05–60 C.


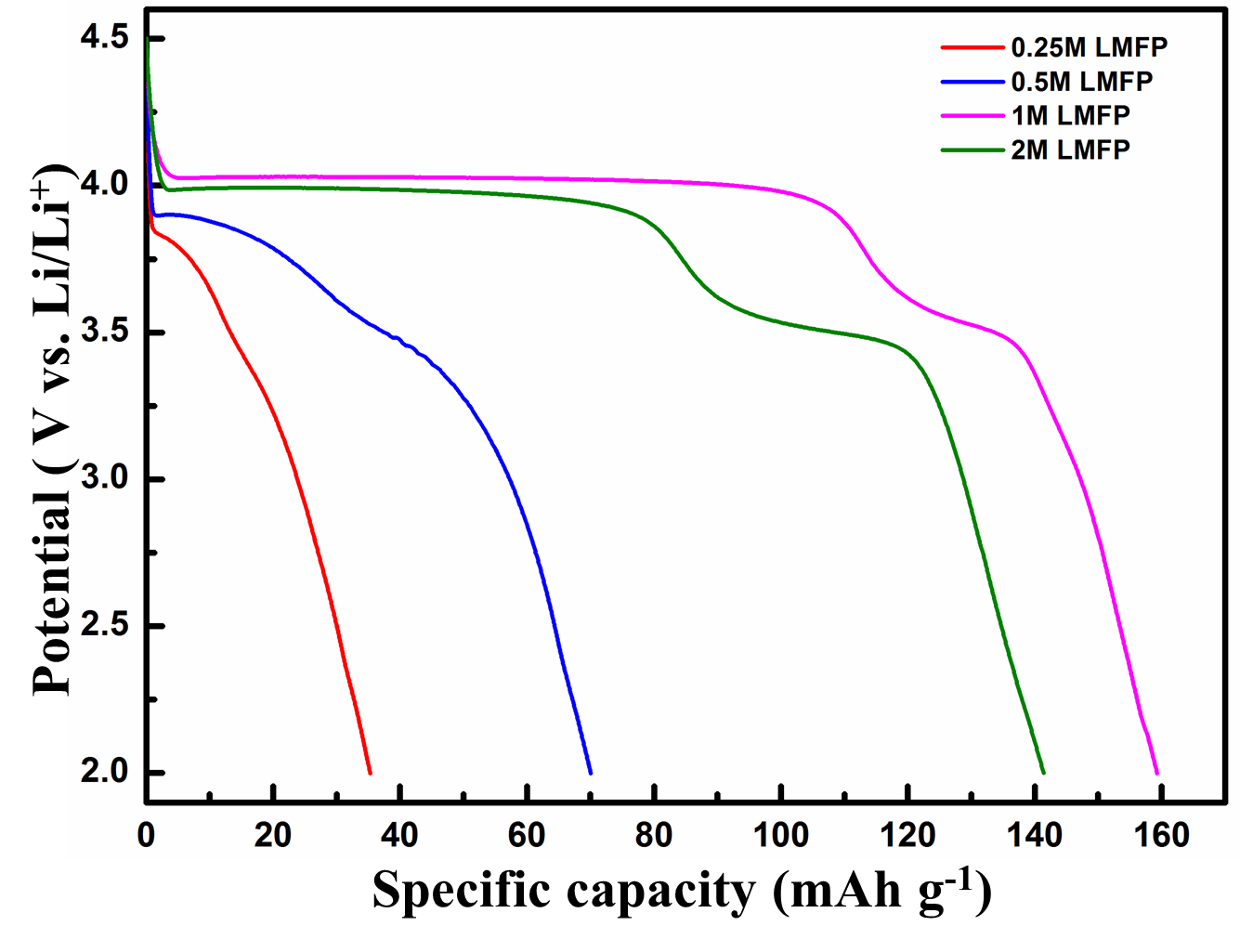


**Figure S8.** Galvanostatic discharge curves of the 3D LiMn_0.75_Fe_0.25_PO_4_/rGO microspheres synthesized using different amounts of the chelating agent at 0.05 C-rate.

**
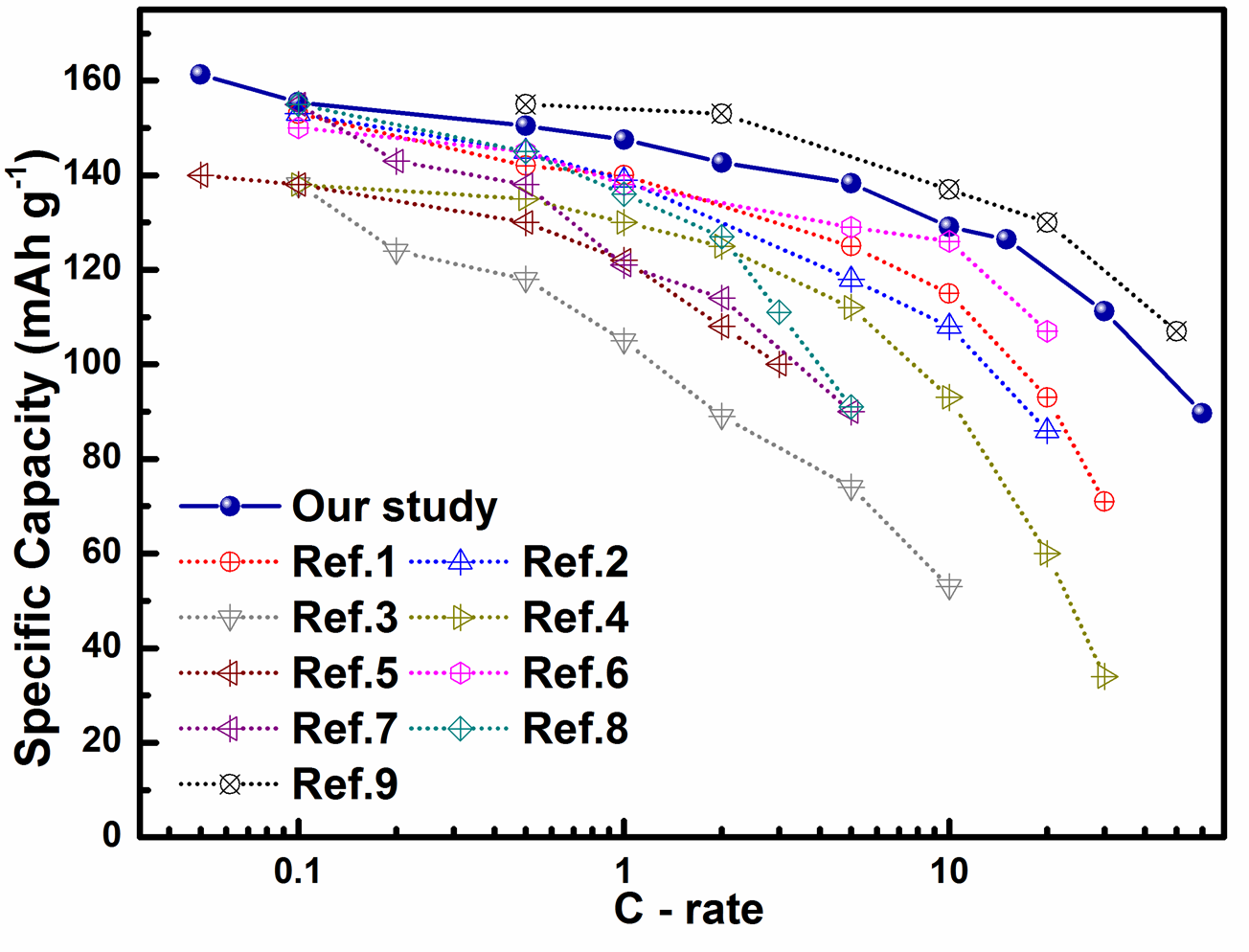
**

**Fig. S9.** Comparison of the rate capabilities (discharge capacity versus discharge rate) of Cl2_LMFP synthesized in this study with those reported for microspherical LiFePO_4_/C[^1-5^](#_ENREF_1), LiMn_x_Fe_1-x_PO_4_/C[^6-8^](#_ENREF_6), and 2D LiMn_x_Fe_1-x_PO_4_/rGO^9^.

The 3D LiMn_0.75_Fe_0.25_PO_4_/rGO microspheres prepared in this study showed a specific capacity of 120 mA h g^-1^ at a high rate (C-rate of 10). This specific capacity is 75% of the specific capacity at a C-rate of 0.05, indicating that the prepared 3D LiMn_0.75_Fe_0.25_PO_4_/rGO microspheres had an excellent high-rate capability. We also compared the rate capabilities (discharge capacity versus discharge rate) of the 3D LiMn_0.75_Fe_0.25_PO_4_/rGO microspheres synthesized in this study with those reported for microspherical LiFePO_4_/C, LiMn_x_Fe_1-x_PO_4_/C, and 2D LiMn_x_Fe_1-x_PO_4_/rGO. The rate capabilities of the 3D LiMn_0.75_Fe_0.25_PO_4_/rGO microspheres synthesized in this study were better than or similar to those of the above-mentioned materials.

**
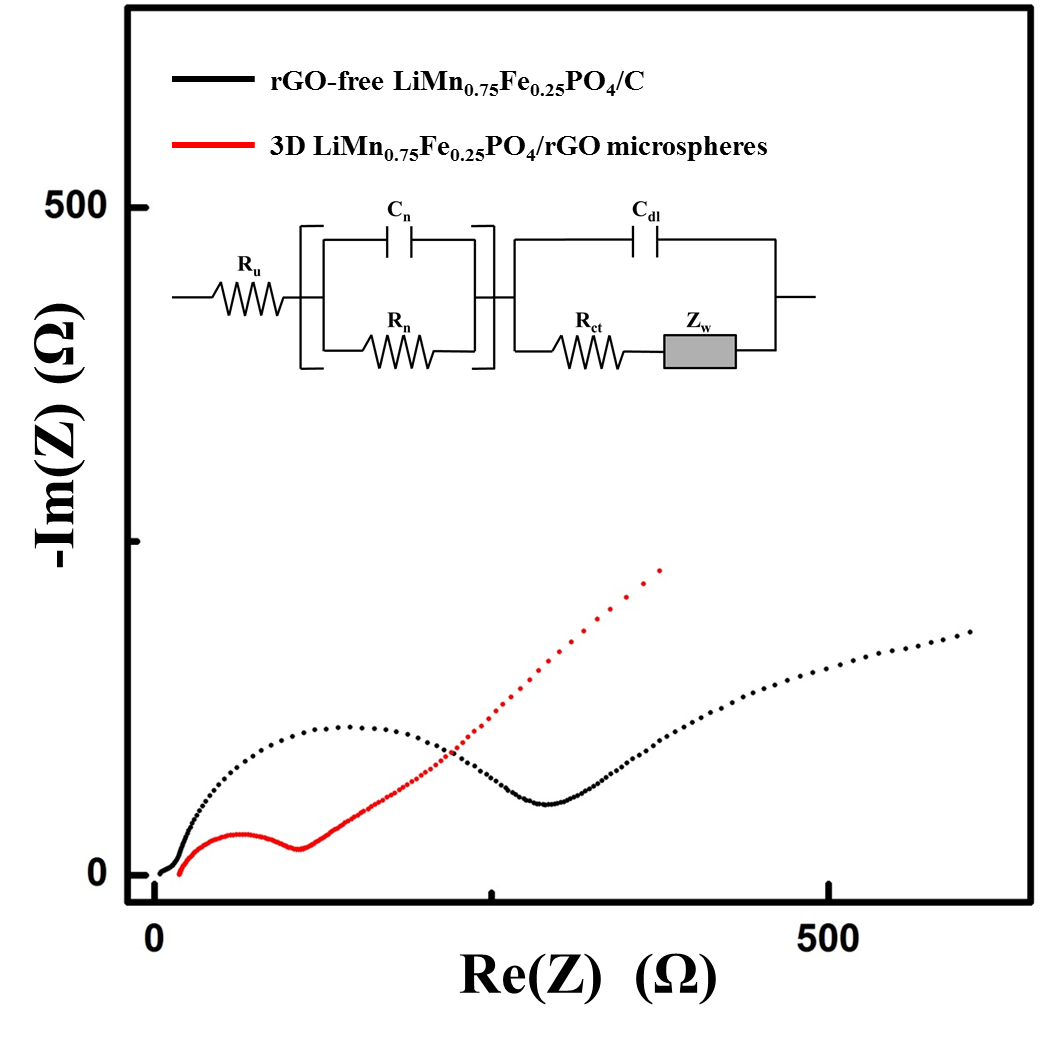
**

**Fig. S10.** Nyquist plots for the rGO-free LiMn_0.75_Fe_0.25_PO_4_/C and Cl2_LMFP.

**References**

[1] J. F. Qian, M. Zhou, Y. L. Cao, X. P. Ai and H. X. Yang, J Phys Chem C, 114 (2010) 3477-3482.

[2] F. Yu, J. J. Zhang, Y. F. Yang and G. Z. Song, J Power Sources, 195 (2010) 6873-6878.

[3] C. W. Sun, S. Rajasekhara, J. B. Goodenough and F. Zhou, J Am Chem Soc, 133 (2011) 2132-2135.

[4] M. Y. Cho, K. B. Kim, J. W. Lee, H. Kim, H. Kim, K. Kang and K. C. Roh, RSC Adv, 3 (2013) 3421-3427.

[5] J. Su, X. L. Wu, C. P. Yang, J. S. Lee, J. Kim and Y. G. Guo, J Phys Chem C, 116 (2012) 5019-5024.

[6] S. M. Oh, S. T. Myung, J. B. Park, B. Scrosati, K. Amine and Y. K. Sun, Angew Chem Int Ed, 51 (2012) 1853-1856.

[7] Y. K. Sun, S. M. Oh, H. K. Park and B. Scrosati, Adv Mater, 23 (2011) 5050-5054.

[8] W. Liu, P. Gao, Y. Y. Mi, J. T. Chen, H. H. Zhou and X. X. Zhang, J Mater Chem A, 1 (2013) 2411-2417.

[9] H. L. Wang, Y. Yang, Y. Y. Liang, L. F. Cui, H. S. Casalongue, Y. G. Li, G. S. Hong, Y. Cui, H. J. Dai, Angew Chem Int Edit, 50 (2011) 7364-7368.
